# Pharmacological Exploration of Phenolic Compound: Raspberry Ketone—Update 2020

**DOI:** 10.3390/plants10071323

**Published:** 2021-06-29

**Authors:** Shailaja Rao, Mallesh Kurakula, Nagarjuna Mamidipalli, Papireddy Tiyyagura, Bhaumik Patel, Ravi Manne

**Affiliations:** 1 Department of Neurosurgery, Stanford University, Stanford, CA 94305, USA; shailajapr.rao@gmail.com; 2 Department of Biomedical Engineering, The University of Memphis, Memphis, TN 38152, USA; 3 Analytical R&D, Akron Inc., Lake Forest, IL 60045, USA; mngarjunrao@gmail.com; 4 Analytical R&D, BioDuro LLC, San Diego, CA 92121, USA; tiyagura.papireddy@gmail.com; 5 Product Development Department, Cure Pharmaceutical Corporation, Los Angeles, CA 90025, USA; Bhaumikp17@gmail.com; 6 Chemtex Environmental Laboratory, Quality Control and Assurance Department, Port Arthur, TX 77642, USA; ravimannemr@gmail.com

**Keywords:** raspberry ketone, hepatoprotection, cardioprotection, gastroprotection, obesity, depigmentation, bone regeneration

## Abstract

Raspberry ketone (RK) is an aromatic phenolic compound naturally occurring in red raspberries, kiwifruit, peaches, and apples and reported for its potential therapeutic and nutraceutical properties. Studies in cells and rodents have suggested an important role for RK in hepatic/cardio/gastric protection and as an anti-hyperlipidemic, anti-obesity, depigmentation, and sexual maturation agent. Raspberry ketone-mediated activation of peroxisome proliferator-activated receptor-α (PPAR-α) stands out as one of its main modes of action. Although rodent studies have demonstrated the efficacious effects of RK, its mechanism remains largely unknown. In spite of a lack of reliable human research, RK is marketed as a health supplement, at very high doses. In this review, we provide a compilation of scientific research that has been conducted so far, assessing the therapeutic properties of RK in several disease conditions as well as inspiring future research before RK can be considered safe and efficacious with limited side effects as an alternative to modern medicines in the treatment of major lifestyle-based diseases.

## 1. Introduction to Raspberry Ketone

Raspberry ketone (RK; 4-(4-hydroxyphenyl) butan-2-one), one of the major aromatic compounds found in European red raspberry (*Rubus idaeus*), defines the characteristic fragrance and taste of raspberry. Raspberry ketone may also be found in other fruits such as peaches, grapes, apples, various berries, vegetables, and in the bark of yew, maple, and pine. Raspberry ketone is widely used in perfumes, cosmetics, and as a flavoring agent in food and beverages [1,2,3]. Although chemically synthesized, RK can be produced via the condensation of *p*-hydroxybenzaldehyde with acetone; it is only considered as a nature-identical flavoring substance according to the EU and US regulations [4] and cannot be marketed as a “natural” flavoring compound according to food laws [5]. The biosynthesis of RK is a two-step process. The first step begins with the phenylpropanoid subsequently hydrolyzed into p-coumaric acid in the raspberry fruit. Coumaroyl-CoA (an intermediate of the lignin synthesis pathway) condensed with one malonyl-CoA to form *p*-hydroxybenzalacetone [4-(4-hydroxyphenyl)-but-3-ene-2-one] by an enzyme called benzalacetone synthases (BAS), a type III polyketide synthase (PKS). The second step is the double bonds of the *p*-hydroxybenzalacetone are reduced by an NADPH-dependent benzalacetone reductase (BAR) and the expense of NADPH to form RK [1,4]. Extraction of raspberry ketone from raspberries or fruits containing RK is very expensive. The main limitations are due to the seasonal availability of fruits as well as the small concentrations of RK naturally occurring in these fruits. To overcome these limitations, RK is currently synthesized chemically via the condensation of p-hydroxybenzaldehyde with acetone or biosynthesized in genetically modified microorganisms such as bacteria or yeast.

The physicochemical properties of RK are given in Table 1. One group produced natural raspberry compounds by cloning the genes involved in the natural synthesis of RK, in micro-organisms [1]. Alternatively, another group described a method to produce biosynthetic RK by converting the precursor, betuloside, in a secondary alcohol dehydrogenase (ADH) producing micro-organisms [6]. A recent report suggests that introducing four heterologous genes, encoding phenylalanine/tyrosine ammonia-lyase, cinnamate-4-hydroxylase, coumarate-CoA ligase, and benzalacetone synthases in an industrial strain of *S. cerevisiae* increased the yields of RK compared to traditional methods [7].

Raspberry ketone has been qualified as a “Generally Reconized As Safe” (GRAS) flavoring agent and has been widely used. Raspberry ketone has also gained a lot of attention as a nutraceutical that owes potential health benefits. Numerous research studies have indicated its anti-obesity, anti-androgenic activity, cardio/hepatoprotective, and anti-inflammatory properties [8].

A study assessing the metabolism of RK in male rats, rabbits, and guinea pigs showed that RK was rapidly absorbed from the gastrointestinal tract after administration of 1 mmol/kg by oral gavage. More than 90% of ingested RK was excreted in urine within 24 h, primarily reduced to raspberry alcohol [9]. Raspberry ketone and its metabolites are bioavailable in both plasma and the brain. Raspberry ketone catabolism is a well-coordinated process involving intracellular ketone reduction and methylation into metabolites including methylated raspberry ketone (RK-Me) and raspberry alcohol (ROH). Of the many health benefits reported, the anti-obesity property of RK has gained the most popularity. Adding RK as a supplement to a weight loss plan is suggested to decrease the accumulation of fats around the abdominal organs and enhance fat burning efficiency [10]. Raspberry ketone has shown to stimulate the metabolism of white and brown adipose tissues via norepinephrine-induced lipolysis, inhibiting the absorption of dietary fat in the small intestine [11,12]. Raspberry ketone has also been reported to possess anti-androgenic [13], depigmentation [14], anti-inflammatory [15] as well as cardioprotective properties [16]. A potential role of RK in Alzheimer’s disease and bone regeneration has also been suggested.

The aim of this review was to discuss the beneficial properties of RK and its underlying mechanisms as well as touch upon available information regarding bioavailability and toxicity.

Electronic databases, such as PubMed, Web of Science, Scopus, and Science Direct, were searched between April 2010 and December 2020 to review the benefits, safety, and potential effects of RK for the treatment or prevention of many lifestyle-based diseases. The scrutiny focused mainly on pre-clinical evaluations reported for RK as a criterion to cite and not the clinical trials, as there were very few or they were negligible. Since it was a non-systematic review, we used keywords such as “raspberry ketone”, “hepatoprotection”, “cardioprotection”, “gastroprotection”, “obesity”, “pigmentation”, and “bone regeneration”.

## 2. Potential Role of RK as a Hepatoprotection

Globally, 844 million people are considered to be affected by chronic liver disease, with a mortality rate of 2 million per year [17]. Most liver diseases are caused due to the fact of viruses (hepatitis A, B, and C), drug or alcohol consumption (alcoholic liver disease), and metabolic liver disease (non-alcoholic fatty liver disease), leading to cirrhosis of the liver and liver failure [18]. The pathophysiology of chronic liver disease involves hepatocyte death, resulting in inflammation and fibrosis, and studies have shown a close relation between liver diseases and metabolic diseases such as obesity, diabetes, and hyperlipidemia [19,20,21]. Apart from a liver transplant, very few treatment options are available to effectively treat and reverse the progression of chronic liver diseases. Dietary and lifestyle changes with a combination of drug therapy are the currently available methods of managing liver diseases like NAFLD. Medicinal plant extracts, such as phytophenols, have proven to be useful in treating several diseases and could be a potential alternative form of treatment or help in slowing the progression of liver diseases.

Raspberry ketone was initially studied for its role as a weight-reducing agent, but recently, more interest has been generated in studying its function in treating other diseases as well. One study demonstrated that intragastric administration of RK could improve the liver condition in high-fat diet-fed rats after four weeks of treatment [22]. Their results show that RK decreased lipids and free fatty acid (FFA) generation in serum and hepatic tissue thus protecting liver cells. The antioxidant activity of RK was demonstrated by the increase in total antioxidant capacity (TAC), an increase in superoxide dismutase (SOD), with a concomitant decrease in the thiobarbituric acid reactive substances (TBARS) level. The study also found peroxisome proliferator-activated receptor-α (PPAR-α) and low-density lipoprotein receptor (LDLR) levels to be lower in NASH rat livers compared to the control rats, which is considered the main cause of disrupted fatty acid metabolism and liver lipidosis. Raspberry ketone treatment was successful in elevating the levels of PPAR-α and LDLR thus suggesting a role for RK as a hepato-protectant and re-establishing the lipid balance. In another study, researchers demonstrated that RK could prevent hepatic damage in rats that were exposed to carbon tetrachloride (CCL4) induced hepatic damage. The CCL4 caused hepatotoxicity by increasing oxidative stress and DNA fragmentation, indicative of increased apoptosis. The CCL4 also induced the expression of inflammatory cytokines such as nuclear factor-κB (NF-κB) and tumor necrosis factor-α (TNF-α). Raspberry ketone was found to effectively reduce oxidative stress and also maintain hepatocyte integrity and microstructure after histological assessment. Raspberry ketone demonstrated antiapoptotic activity by reducing cytoplasmic expression of cytochrome C and caspases and also by inhibiting DNA fragmentation. Interestingly, in this study, pretreatment with RK inhibited a CCL4-induced increase in inflammatory markers thus arguing for its use as a prophylactic to reduce the impact of hepatic diseases [23].

Another group similarly demonstrated that RK in combination with white tea could protect rats from acrylamide hepatotoxicity by functioning as an anti-inflammatory, antioxidant, and anti-apoptotic agent. The combination treatment of RK and white tea, after acrylamide-induced liver damage, showed a decrease in elevated liver enzymes such as aspartate aminotransferase (AST), alanine aminotransferase (ALT), and alkaline phosphatase (ALP). Immuno-histological examination of rat livers showed a reduction in hepatic damage, measured as a decrease in necrotic changes and reduced inflammation and vacuolation [24].

**Figure 1:**
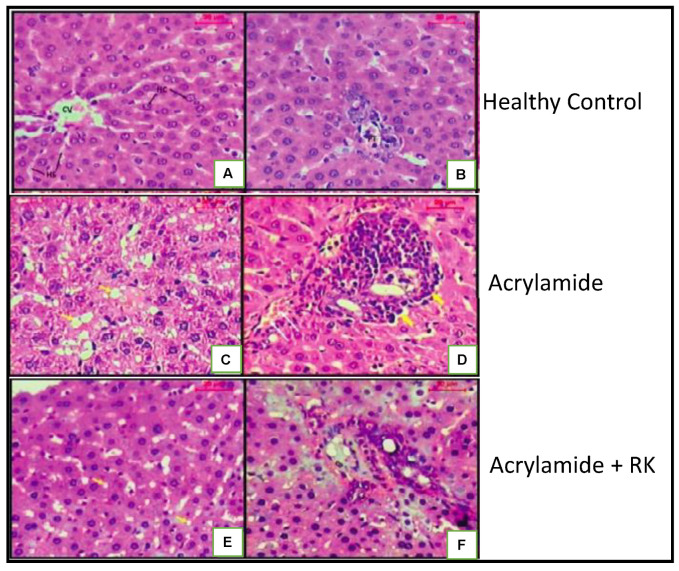
RK-mediated hepatic protection in rats after acrylamide-induced hepatic disease. A micrograph of a liver section of a healthy control (**A**,**B**), showing the normal architecture of a hepatic lobule. The central vein is surrounded by hepatocytes, hepatic sinusoids, and the normal portal tract. (**C**,**D**) The liver sections of the acrylamide-treated group showing the disturbance of the hepatic lobules, vacuoles in the hepatocytes, massive lymphocyte infiltration in the portal, and periportal spaces with dilated and congested veins. (**E**,**F**) The RK-acrylamide-treated group showing the hepatic lobule that appeared more or less like normal; the portal tract associated with mild infiltration and hepatocytes appeared almost normal. H&E staining; scale bar: 20 mm. Adapted from [24].

The mechanism of action of RK is still unclear but based on the limited animal studies that have been conducted, it can be surmised that RK, when consumed as a dietary supplement, could protect the liver in several ways based on the type of trigger leading to liver damage. High-fat diet-induced steatohepatitis in rats was reversed by RK, mainly by reducing fat deposits in the liver cells as well as decreasing apoptosis and degeneration of the liver cells. Raspberry ketone treatment helped improve leptin resistance, reduce the release of TNF-α, increase superoxide dismutase (SOD) activity, and decrease malondialdehyde (MDA) levels as well as adjust fatty acid metabolism by increasing PPAR-α receptors and LDLR expression [22]. It is speculated that acrylamide-induced liver toxicity could be improved after RK and white tea treatment by increasing levels of SOD and catalase, which helps in clearing out free radicals and preventing oxidative stress (Figure 1). Raspberry ketone and white tea treatment also led to increased adiponectin levels and a decrease in caspase-3, an apoptosis marker level. Histopathological studies showed concurrent improvement in hepatic cell structure and function [24]. The CCL4-induced liver toxicity could be similarly reversed by RK treatment, thus strengthening the evidence for RK to be considered as a possible treatment for acute and chronic liver diseases. The current studies indicate that RK improves liver disease in mice by elevating PPAR-α expression.

**Figure 2:**
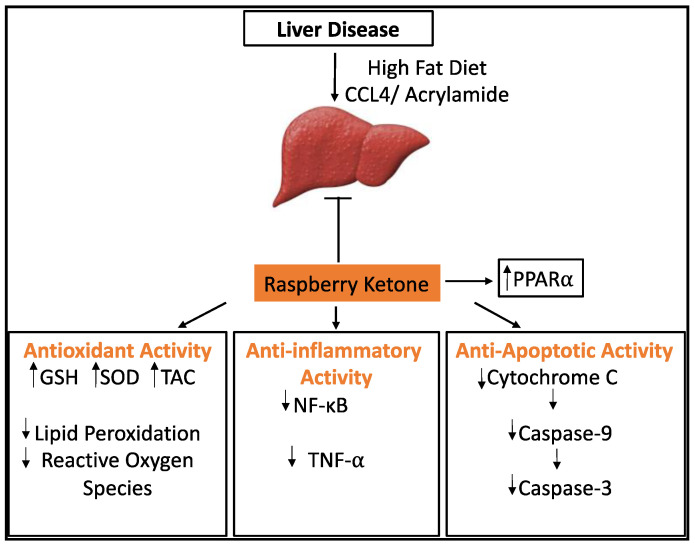
Schematic diagram showing the protective effects of RK against liver disease caused by a high-fat diet, CCL4, or acrylamide.

This result needs further validation in different liver disease models, and for RK to be considered as a therapy, highly detailed animal experimentation needs to be performed to assess the exact mechanisms and signaling pathways that are activated by different doses of RK within the therapeutic window, followed by rigorous clinical studies. The possible mechanism of RK as a hepatoprotective agent is summarized in Figure 2.

Raspberry ketone treatment was able to reverse liver damage in rodents by its anti-oxidant, anti-inflammatory, and anti-apoptotic activity. It is speculated that most of these effects are due to the fact of its ability to increase the expression of PPAR-α.

## 3. Potential Role of RK as a Cardioprotectant

Cardiovascular diseases (CVDs) are the leading cause of mortality worldwide. They mainly include atherosclerosis, hypertension, cardiac hypertrophy, myocardial infarction, and heart failure [25]. Myocardial infarction (MI), or a heart attack, occurs due to the fact of cardiac ischemia. Occlusion of coronary arteries decreases the oxygen supply to cardiac cells, causing necrosis of the cells, leading to MI. Intracellular oxygen levels are of significant importance to the normal functioning of cardiac cells [26]. Cardiac cell necrosis due to the reduced oxygen levels leads to deleterious effects, leading to the loss of cell membrane integrity and an uncontrolled release of intracellular content into extracellular space [27]. The most important effect of cardiac cell necrosis is the development of cardiac arrhythmias due to the loss of functional contractile muscle mass and electrolyte imbalance caused by cell lysis [28]. Myocardial infarction progresses into heart failure eventually, due to the overactivation of the neurohumoral system, increased inflammatory response, oxidative stress, disordered cardiac growth, fibrosis deposition, altered gene/protein synthesis, and energy starvation. The chronic dysregulation of these systems leads to heart failure causing congestion and pulmonary edema leading to incapacitation and poor outcomes. Teasing out the mechanisms leading to heart failure due to the fact of MI is an essential step in the development of new therapies. The most commonly used treatment options for CVDs are angiotensin-converting enzyme (ACE) inhibitors, triglyceride-lowering agents, anti-platelet agents, aspirin, nitroglycerine, and beta-blockers. Despite great strides being made in the diagnosis and treatment of CVDs, it remains a leading cause of mortality in most developed countries, thus calling for more innovative strategies for prevention and treatment [29].

Several herbs and phytoconstituents have been studied for their role as cardio protectants. One group studied the effect of RK on isoproterenol (ISO) induced MI. ISO-induced MI in rodents is a well-accepted strategy to study MI as it causes severe oxidative stress in the myocardium, which is considered the main mechanism of myocardial necrosis. In Wistar rats with ISO-induced MI, they found increased oxidative stress due to the fact of ROS and also an increase in MDA levels which led to a decrease in antioxidant levels of superoxide dismutase (SOD), catalase (CAT), and glutathione (GSH), dysregulated lipid metabolism, and inflammation due to an increase in reactive nitrogen species. The histological sections of ISO-treated rats showed extensive myocardial damage with edema, disrupted cardiac myofibrils, inflammatory cells, and necrosis of ventricular regions. The study found that treating rats with 100 mg/ka and 200 mg/kg of RK could reverse the levels of ROS enzymes, which could be attributed to an increase in PPAR-α expression [30]. Raspberry ketone was able to normalize lipid parameters, reduce peroxynitrite levels, and normalize myocardial tissue architecture. The study speculates that these effects were mainly brought about by the ability of RK to increase the expression of PPAR-α. It is speculated that PPAR-α functions by binding to PPAR-responsive elements (PPREs), which have been identified in the promoter regions of several antioxidant, anti-inflammatory, and lipid homeostasis genes [31]. A follow-up study by the same group demonstrated that RK has an affinity to bind PPAR-α, by docking analysis. While they observed an increase in PPAR-α levels in the presence of RK, evidence of direct binding between the two would greatly strengthen the role of RK as a cardioprotectant [32].

**Figure 3:**
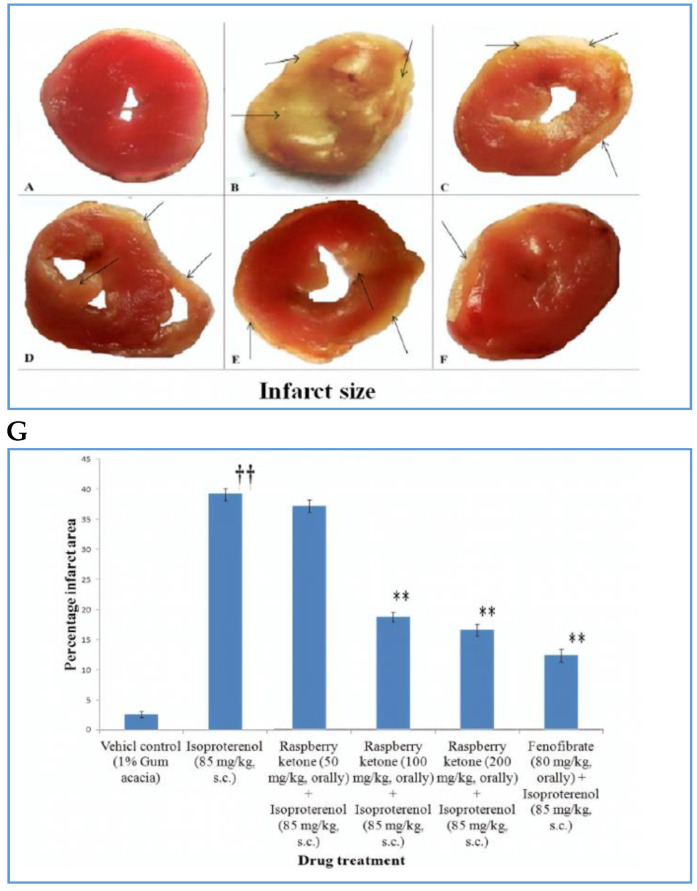
Representative figures showing the effect of RK and different drugs on the myocardial infarct size of various groups (shown with arrows). (**A**) Vehicle control (1% gum acacia), (**B**) isoproterenol (85 mg/kg, s.c.), (**C**) RK (50 mg/kg, orally) +isoproterenol (85 mg/kg, s.c.), (**D**) RK (100 mg/kg, orally) +isoproterenol (85 mg/kg, s.c.), (**E**) RK (200 mg/kg, orally) + isoproterenol (85 mg/kg, s.c.), and (**F**) fenofibrate (80 mg/kg, orally) +isoproterenol (85 mg/kg, s.c.). The graph shows the percentage infarct area of different treatment groups (**G**). Each column represents the mean SEM. Significance was determined by one-way ANOVA followed by Dunnett’s t-test: ** *p* < 0.01 versus toxic control; †† *p* < 0.01 versus vehicle control. Adapted from [16].

Figure 3 shows that pre-treatment with RK was able to protect rats from MI similar to fenofibrate, a classic drug used for the treatment of MI. Two studies on rats [16,30] provide convincing evidence of the potential use of RK as a treatment strategy for MI, but more work on animals and clinical studies need to be performed to confirm its therapeutic potential.

## 4. Potential Role of RK in the Treatment of Gastric Ulcers

Gastric ulcers are the most common disorders of the gastrointestinal tract, affecting approximately 2.4% of the western population [33]. A higher incidence of gastric ulcers is observed in people who are infected with Helicobacter Pylori (H. Pylori), who smoke, consume alcohol, and use nonsteroidal anti-inflammatory drugs (NSAIDs) [34,35]. In a very small population with H. Pylori infection or who are taking NSAIDs develop peptic ulcers, implying that there could be other biological or environmental factors that are essential for the initiation of mucosal damage. The method by which H. Pylori causes mucosal damage is still unknown, but it is hypothesized that H. Pylori produces bacterial factors that interact with the host tissue, causing gastric mucosal inflammatory cell infiltration and gastric epithelial injury [34]. Nonsteroidal anti-inflammatory drugs are known to damage the mucosal lining by disrupting mucosal phospholipids and uncoupling mitochondrial oxidative phosphorylation. The presence of gastric contents, such as acid, pepsin, food, and bile, further damage the mucosa [35,36]. Reduced cyclooxygenase 1 (COX-1)-derived prostaglandin levels are also considered an important mechanism leading to mucosal damage [37]. The main complications of peptic ulcers are bleeding, perforation, and gastric outlet obstruction [38]. Reducing acid secretion or reducing gastric-related stress factors is the main aim of treatment for peptic ulcers. Over the past decades, hospital admissions for peptic ulcers have fallen, but the mortality rate still is approximately 5–10% [39]. Although some drugs are available for the treatment of gastric ulcers, they also cause some side effects or fail to treat the condition. Plant-based drugs have been extensively studied for the treatment of gastric ulcers and of these, hesperidin and quercetin have shown to be effective [40]. Plant-based drugs are considered to cause fewer side effects and, thus, could have a higher efficacy compared to conventional medicine.

**Figure 4:**
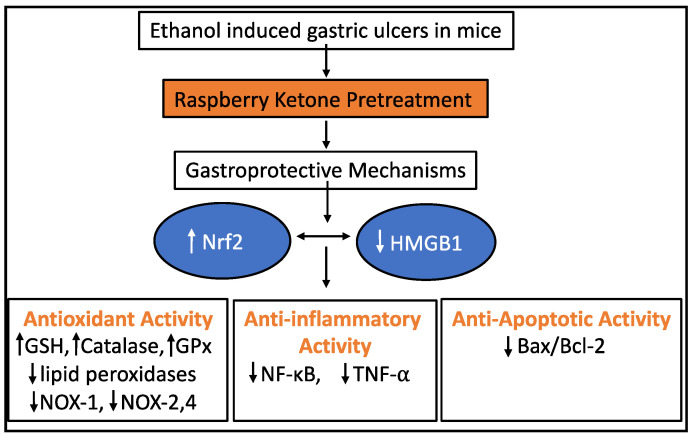
Schematic diagram of the different molecular targets involved in the gastroprotective effects of RK against ethanol-induced gastric ulcer in rats.

Raspberry ketone was studied for its ability to protect Wistar rats from gastric ulceration. Ethanol is a common cause of gastric ulceration through increasing oxidative stress. NADPH oxidases (NOXs) and Nuclear factor erythroid 2-related factor 2 (Nrf2) play an important role in ethanol-induced gastric ulcers. The study used ethanol-induced gastric ulceration in mice as a model to test the effect of RK. They found that ethanol-induced gastric ulcer was associated with Nrf2 downregulation and, at the same time, caused the upregulation of NOX-1, 2, and 4 and high-mobility group box-1 (HMGB1), a nucleoprotein that mediates inflammatory functions. These changes were reversed by pretreatment by RK. They further found that gastroprotective properties of RK were mediated by suppression of NF-kB and TNF-α, and reduction of the Bax/Bcl2 ratio [41]. Omeprazole is a commonly used drug in the treatment of gastric ulcers. Omeprazole inhibits the parietal cell H+/K+ ATP pump, thereby suppressing gastric basal and stimulated acid secretion [42]. In the same study, the gastroprotective action of RK was compared to that of omeprazole. Raspberry ketone was found to have a higher gastric protective effect than omeprazole as quantified by the histology of the rat gastric mucosa. This provides strong evidence of a possible role for RK as an anti-ulcer agent (Figure 4). Further studies with different gastric ulcer rodent models are necessary to validate the mechanism as well as find different signaling pathways that might be activated by pre-treatment and to treat pathologic gastric ulcers using RK. Raspberry ketone ameliorated ethanol-induced oxidative stress and suppressed inflammation and apoptosis. The upregulatory effect on Nrf2 and downregulation of NOXs and HMGB1, and their crosstalk play a role in the gastroprotection afforded by RK. Adapted from [41].

## 5. Studies Exploring the Depigmenting Activity of RK

**Figure 5:**
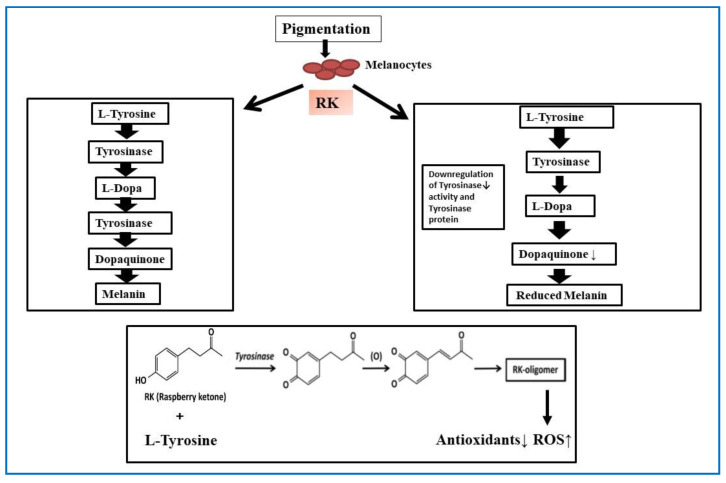
The schematic diagram showing the production of melanin from melanocytes and the effect of RK on melanin synthesis resulting in depigmentation.

The skin color of animals and humans is determined mainly by the content of melanin pigment in the skin. Melanin pigmentation is regulated by melanosome structure and trafficking, melanin synthesis, and melanocyte-specific transcription factors. Tyrosinase (TYR), tyrosinase-related protein 1 (TYRP1), and melanocortin1 receptor (MCIR) have been associated with pigmentation disorders, such as hyperpigmentation, hypopigmentation, and mixed hyper-/hypopigmentation disorders, which can be detected by size, site of the involvement, and patterns of the lesions. Pigmentation disorders are further subclassified into congenital and acquired disorders. Melanin is synthesized in dermal melanocytes by a process called melanogenesis, which is initiated within melanocytes, the melanosomes [43]. Melanin production begins with the oxidation of L-tyrosine to L-DOPA (l-3,4-dihydroxyphenylalanine) and dopaquinone; with the help of tyrosinase. The tyrosinase reactions are considered the rate-limiting process in melanin synthesis; the reaction proceeds spontaneously at physiological pH. Although melanin primarily provides a photoprotective function, the abnormal accumulation of melanin in different parts of the skin can become a problem that results in pigmented skin patches [44,45]. While various natural anti-oxidants have been explored for treating pigmentation conditions, RK has gained importance for the same Figure 5.

Figure 5 scheme explains the RK binding to L-tyrosine forming an RK-oligomer, increasing the ROS levels. One group investigated the role of RK in inhibiting the melanogenesis both in vitro in cultivated murine B16 melanoma cells and in vivo in zebrafish and mice. They found that RK inhibited the melanogenesis by post-transcriptional regulation of tyrosinase gene expression, which resulted in the downregulation of both cellular tyrosinase activity and the amount of tyrosinase protein unaffecting the tyrosinase mRNA transcription levels in B16 cells. Similarly, in zebrafish, RK also inhibited melanogenesis by reducing tyrosinase activity. In the same study, topical application of RK (0.2% or 2%) gel showed skin whitening within one week of treatment, indicating its potential in the cosmetic industry as well [3]. A biomimetic study revealed that exposure to RK may lead to chemical/occupational leukoderma. They identified that RK is structurally similar to 4-[4-hydroxyphenyl)-2-butanol (rhododendrol), which is a skin whitening agent. Rhododendrol is reported to cause tyrosinase-dependent cytotoxicity to melanocytes, and RK might competitively bind, exerting cytotoxicity through tyrosinase-catalyzed oxidation to cytotoxic o-quinones as RK-quinone and DBL-quinone. Both have different reactivities based on their half-lives. DBL-quinones have larger half-life decays to form RK-oligomers that can oxidize reduced glutathione (GSH) to oxidized (GSSG) with the concomitant production of hydrogen peroxide, indicating its pro-oxidant activity. Further, the study confirmed the RK melanocytic cytotoxicity by binding of RK-derived quinones to thiol proteins [46].

## 6. Potential Role of RK as an Anti-Obesity Agent

Obesity has become a major health issue in recent decades, as it is linked to a number of diseases like atherosclerosis, stroke, type 2 diabetes, and certain cancers. About one-third of the adult population in the USA are obese, and the number continues to rise. Unhealthy eating habits, lack of exercise, and socioeconomic status are considered the main drivers of obesity. The interactions between the environment and our genes, resulting in epigenetic changes, are also being considered as possible causes of obesity [47]. The pathophysiology of obesity involves mechanisms such as impaired neuro circuit regulation and neuroendocrine hormone dysfunction. A balanced diet and exercise are the mainstays for treating obesity [48]. Obesity is a leading cause of the metabolic syndrome, a term encompassing several conditions, such as hypertension, central adiposity, hyperglycemia, and dyslipidemia, thus increasing the risk of cardiovascular diseases [49]. The function of adipose tissue goes beyond being an energy storage organ, it is also an endocrine organ secreting bioactive substances. Adipose tissue can secrete pro- and anti-inflammatory substances, and the imbalance between the two contributes to the pathogenesis of metabolic dysfunction [50]. In order to study anti-obesity agents in vitro and in vivo, Wang et al. [51] reviewed the most studied mechanisms for preventing obesity using polyphenols. The mechanisms included a decrease in food intake, lipogenesis, oxidative stress, inflammation, inhibition of adipocyte differentiation, and a concurrent increase in lipolysis, and fatty acid oxidation. Hyperlipidemia is a medical condition, predisposing individuals to major vascular complications and is characterized by an increase in plasma lipids such as triglycerides, cholesterol esters, and phospholipids. Previous reports in rodents showed that prolonged feeding with a high-fat diet increased the adipocyte amount and body size [52]. The current available marketed drugs used to treat hyperlipidemia often resulted in adverse effects on prolonged administration. However, natural phenolic compounds available in the regular diet have indicated promising effects in lowering lipid levels in the body [53].

**Figure 6:**
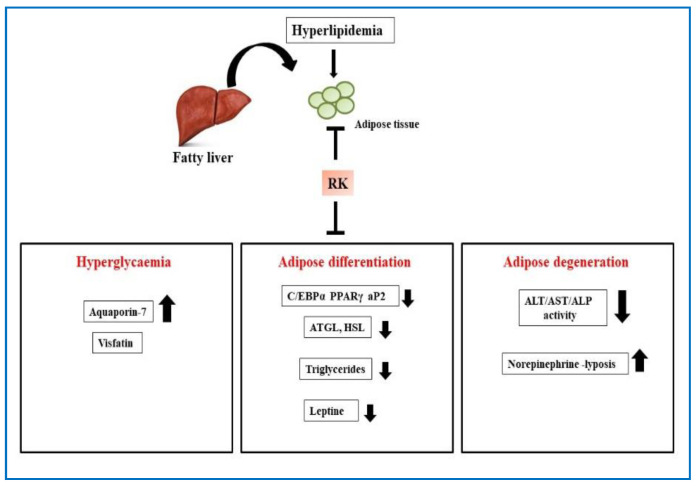
Schematic diagram showing the effect of RK on hyperlipidemia.

An early study by one research group explored the role of RK as an anti-obesity agent in mice. Mice fed with a high-fat diet were given different concentrations of RK mixed with food, followed by measuring body weights, serum lipid parameters, and lipolysis of white adipocytes. The study found a partial dose-dependent decrease in total body weight, as well as weights of the liver and adipose tissues of mice fed with a high-fat diet for 10 weeks, with the greatest decrease with a 2% dose of RK. The mice also showed a partial decrease in hepatic triacylglycerol levels as well as increased lipolysis in response to norepinephrine in white adipose cells in vitro. This early study gave some indications towards the weight-reducing and lipolysis effects of RK [10]. Another group studied the effect of RK on the expression and secretion of adiponectin, lipolysis, and fatty acid oxidation in 3T3-L1 mouse adipose cells. The study found that treatment with 10 μM RK produced an increase in lipolysis, fatty acid oxidation, and expression, secretion of adiponectin and adipocytokine, as well as a reduced lipid accumulation [5]. In a follow-up study to dissect the mechanism of RK, the same group studied the effect of RK on adipogenic and lipogenic gene expression in 3T3-L1 cells. The study found that RK could inhibit the expression of adipogenic and lipogenic genes as well as enhance the expression of genes involved in lipolysis and fatty acid oxidation pathways. They also found that RK suppresses adipogenic differentiation of 2T3-L1 pre-adipocytes by reducing the expression of enhancer-binding protein-α (C/EBPα), peroxisome proliferator-activated receptor-γ (PPARγ), and adipocyte fatty acid-binding protein-2 (aP2) [54]. Raspberry ketone was also found to upregulate the expression of genes involved in lipolysis, fatty acid oxidation pathways including hormone-sensitive lipase (HSL) and adipose triglyceride lipase (ATGL), which play a critical role in triglycerides catabolism [55] Figure 6.

Raspberry ketone-mediated adipose degeneration in the liver acts by decreasing the activities of alanine aminotransferase (ALT), aspartate aminotransferase (AST), and alkaline phosphatase in a model control group rat serum. Raspberry ketone also reduced fat deposition in liver cells by promoting cytostasis to decrease the necrosis of liver cells [22]. In a recent study, aquaporin-7 (AQP7) was identified as an adipocyte-specific glycerol channel correlated with the expression of various adipocytokines that control lipid homeostasis. To identify the potential of RK in controlling body weight gain, hyperlipidemia, and insulin resistance in male obese rats, one group administered low and high doses of RK on high-fat (HF) diet-fed rats and found that both doses effectively upregulated AQP7 and downregulated leptin expression, which greatly helped to control lipid levels [11]. The study demonstrated in mice that, RK treatment after a high-fat diet reduced adiposity gain, decreased ghrelin levels, and hepatic metabolic markers. Further studies are still required to elucidate the pathways leading to the anti-obesity function of RK. Interestingly, a study conducted to identify the adverse effect of RK in obese and health-compromised obese mice, revealed that mice fed with 330 mg/kg RK showed retardation of gain in body weights compared to control groups. Higher doses of RK at 500 mg/kg indicated high levels of blood glucose and ALT. This study even indicated that RK can cause adverse effects in health compromised conditions and high doses may pose a potential risk [56].

The bioavailability and metabolism of RK in mice were studied using a targeted metabolomic approach in order to evaluate the route of administration and the efficacious dose required. The results from this study found that high-fat diet-induced obese mice have a higher bioavailability and absorption of RK, and RK also accumulated in lipid-rich tissues such as the brain. The results from the in vitro and mouse studies strengthen the claims of RK being a potential anti-obesity agent [57]. A single clinical study showed that a multi-ingredient supplement containing RK was able to reduce body weight, metabolic lipid, and inflammatory parameters in 45 obese individuals after an 8 week exercise and weight loss program. Although the supplement consisted of different concentrations of other herbs in addition to RK, including, caffeine anhydrous, bitter orange fruit, ginger root extract, garlic root extract, cayenne extract, L-theanine, and pepper extract, it is feasible to assume that at least a certain extent of weight lowering effect was due to the addition of RK. The combination of exercise, prescribed diet, and supplement intervention led to positive weight loss benefits [58]. More clinical trials with RK as a single supplement should be performed to remove the effect of confounders as well as to determine the best dose of RK for its anti-obesity effects.

## 7. Potential Role of RK in Early Sexual Maturation

Sexual development takes place during the adolescent phase. Most of the methodological surveys have identified that socio-economic, urbanization, nutrition, and other environmental factors have a direct relationship with sexual maturation, obesity, and thyroid system function that can greatly impact human growth and maturation [59]. One study has put forth a hypothesis claiming that early maturation in girls is majorly due to the high levels of internalizing problems that were attributed to their high sensitivity to interpersonal stress. The study even reported that a girl’s cortisol reactivity to interpersonal challenges is more strongly associated with internalizing problems than a boy’s reactivity [60]. Obesity is one of the factors in sexual hormonal imbalance that may result in delayed sexual maturation. Obesity can increase male infertility or subfertility by converting androgens into estrogens in redundant adipose tissue, promoting hypogonadism [61].

Sexual maturation or puberty in humans is associated with the growth and maturation of gonads of the internal sex organs, making the external genitalia occur and the secondary sexual characteristic to develop. The maturation of the adrenal cortex with increased secretion of adrenal androgens is generally observed between age group between 6 and 8 in girls and 7 and 9 in boys. Puberty eventually leads to important periods in both males and females: (i) sexual differentiation during fetal life and (ii) the postnatal surge of gonadotropins. These events are followed by a hypothalamic–pituitary–gonadal axis activation that usually occurs at 2 and 6 months in infant boys and girls, respectively. The biochemical hallmark of adrenarche is in the rise of plasma dehydroepiandrosterone (DHEA), its sulfate, and Δ^4^-androstenedione [62,63].

**Figure 7:**
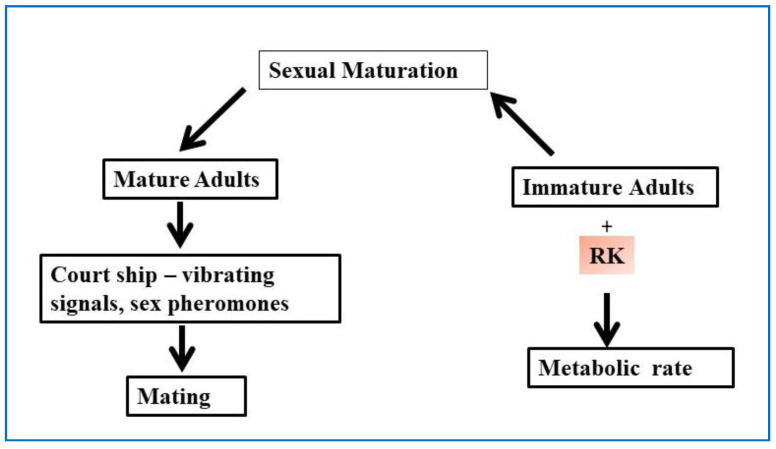
Schematic diagram showing the process of mating in insects and the role of RK in sexual maturation.

Shelly Todd summarized the potential role of RK as pheromone precursors or components that are responsible for sexual communication in Bactrocera species. The study found that females showed preferential attraction to males that were fed RK, also RK helped in boosting the male mating success ratio among the control [64]. Another group conducted a series of experiments to identify RK’s role as a metabolic enhancer; including RK in the diet of male Qflies may accelerate sexual maturation Figure 7. The results concluded that feeding RK along with yeast hydrolysate to immature Qflies increases the mating propensity at young ages [65].

## 8. Potential Role of RK in Bone Regeneration

Bone is a vascularized connective tissue. It has a honeycomb-like matrix internally, giving it rigidity. Bone tissue is subject to continuous remodeling during adult life, and it also has a physiologic process during fracture healing. Bone tissue is made of different types of bone cells. Osteoblasts and osteocytes mineralize the bones, osteoclasts reabsorb bone tissue, other tissues found in bones are bone marrow, endosteum, periosteum, nerves, blood vessels, and cartilage. Although bone regeneration is a physiological process, certain pathological conditions require bone regeneration in large quantities such as in the case of large bone defects, necrosis of bone head due to the reduced blood supply, or genetic defects of the connective tissue making bones fragile [66]. The study of signaling molecules involved in healing fractures is a promising method to find novel therapies for bone regeneration. The main factors to consider while evaluating signaling molecules are to assess their ability to directly influence bone-formation pathways or that induce the production of bone-forming progenitor cells. The signaling molecules that influence bone regeneration can be classified into three broad groups: (i) pro-inflammatory cytokines, (ii) transforming growth factor-β (TGF-β) superfamily and other growth factors, and (iii) angiogenic factors. Bone morphogenic proteins (BMPs) belong to the TGF-β superfamily and are known to play an important role in osteogenesis. Of the BMPs, BMP-2, BMP-6, and BMP-9 were found to be the most effective inducers of osteoblast differentiation of mesenchymal progenitor cells [67]. The different TGF-β’s, platelet-derived growth factors (PDGFs), fibroblast growth factor (FGF), insulin-like growth factors (IGFs), matrix metalloproteinases (MMPs), and other angiogenic factors are all known to have important roles in bone regeneration [68]. The current strategies used for bone regeneration face some limitations related to their use and availability as well as controversy about efficacy and cost-effectiveness. Synthetic bone substitutes generated do not match the biological and mechanical properties of the bone. Hence, it is necessary to develop novel treatment alternatives to overcome these limitations [69]. Raspberry ketone, an organic polyphenol, could potentially overcome the limitations of synthetic substitutes and may aid osteogenesis.

A single study demonstrated the effect of RK in inducing differentiation of mesenchymal stem cells into osteoblasts or adipocytes. The authors examined the effect of RK on osteoblast differentiation on C3H10T1/2 stem cells and mesenchymal stem cells derived from mouse primary bone marrow stromal cells. Osteoblast differentiation from C3H10T1/2 was initiated by using all-trans-retinoic acid (ATRA) and recombinant human bone morphogenic protein-2 (rhBMP-2) [70]. Both ATRA and BMP-2 are known to play roles in osteoblast differentiation as well as increase alkaline phosphatase (ALP) activity, an early osteoblast marker in C3H10T1/2 cells [71,72,73]. As osteoblast generation and adipogenesis are reciprocal processes, the authors examined the effect of RK on adipogenesis in their cells. The authors found that RK could induce C3H10T1/2 stem cells to differentiate into osteoblasts. They also demonstrated that osteoblast differentiation by RK might not be mediated by TGF-βs. Raspberry ketone was also capable of early-stage adipocyte differentiation as measured by elevated TG levels in the cells, but late-stage cells showed a decrease in TG levels [70]. Although the study provides some evidence of an osteogenic role of RK to be considered as a therapy, substantial animal experimentation needs to be performed. In mice, RK-treated mesenchymal stem cells could be implanted into a tibia defect model to assess bone regeneration by these cells. Ectopic bone formation in mice can be assessed by growing cells on scaffolds, followed by implantation into mice and assessing new bone formation after 6–8 weeks. Systemic administration of RK as a supplement can also be given to mice with bone defects to study its effect on bone regeneration [74]. It would be an interesting aspect and a strong basis if the osteogenic potential of RK is examined using murine stromal cell lines (W20-17) or mouse osteoblast precursor cell lines (MC3T3) that can provide valuable data. In addition, pro- or anti-inflammatory modulation of RK needs to be more carefully examined in human macrophage cell lines (RAW 263). This can be beneficial in the cases of orthopedic surgeries especially in the case of metal or biomaterial-based implants. The RK can be combined with biomedical devices and can be locally released to inhibit the cascade of inflammatory mediators at the site of implantation. The review summaries the therapeutic properties of raspberry ketone along with mechanism of action in Table 2.

## 9. Pharmacokinetic (ADME) and Safety Profile of RK

Recently, RK has received a lot of attention due to the fact of its potential advantages as an anti-obesity, anti-inflammatory, anti-apoptotic, and gastric protectant agent. Raspberry ketone is used as a dietary supplement, most commonly as an anti-obesity agent. Despite its use as a dietary supplement, very little is known about its metabolism, bioavailability, and safety. It is imperative to know the pharmacokinetics and toxicity of substances to ensure their suitability for human consumption at doses recommended in commercial products [8]. One research group carried out two pharmacokinetic studies to assess the bioavailability and metabolism of RK in mice of the opposite sex and obese vs. normal mice. They found RK to be highly bioavailable orally, rapidly metabolizing, and to have a lipid-lowering property in obese by accumulating in lipid-rich tissues such as the brain and white adipose tissue. In normal-weight mice, RK and most of its metabolites were detected in plasma within 30 min and returned to baseline at 6 h. Obese mice demonstrated a higher capacity to absorb and retain RK and some of its metabolites, resulting in higher concentrations and longer elimination half-lives of metabolites. Due to the high bioavailability, it is suggested that a much lower dose might be suitable instead of the much higher doses that are currently advertised [12]. A very recent publication describes the development and validation of ultra-high-performance liquid chromatography with a triple quadrupole tandem mass spectrometry (UHPLC-QqQ-MS/MS) method for targeted determination of RK and its metabolites in plasma and brain specimens in mice. They believe that based on this method, it would be possible to study the pharmacokinetics and toxicity of RK as well as establish the safest effective dose that can be consumed for human use [57]. A pioneering study to assess the safety and toxicity implications of consuming RK as a dietary supplement generated a quantitative structure–activity relationship (QSAR) model that predicts the activity of new chemicals. The study predicted possible adverse effects of RK on reproduction and cardiac functions. However, at this time there are no in vivo studies to address these possible adverse effects with RK. Considering the very high recommended daily doses between 100 and 1400 mg, it is vital to evaluate the hazards that may occur with daily supplementation of RK [16,75,76].

## 10. Conclusions

There has been an increased interest worldwide in the study of nutraceuticals, in the pharmacological exploration of bioactive ingredients, and their potential to treat diverse diseases. Preliminary in vitro and in vivo animal studies have shown beneficial effects of RK in hepatoprotection, cardioprotection, and in the treatment of gastric ulcers and obesity, to name a few. For RK to be considered as a therapeutic molecule, however, extensive studies need to be carried out to establish its efficacy and mechanism of action. Admittedly, in the future perspective, RK can be considered as a lead moiety, identified and regarded as a versatile phytoconstituent, having a plethora of therapeutic and nutritional values, but more rigorous safety and toxicological studies are required before raspberry ketone can be considered for disease treatment in first line. We expect the present comprehensive review on RK will serve as baseline data to encourage further detailed investigations on this key biomolecule and would further enable researchers to explore and unwind other properties of RK that can aid the unmet clinical needs.

## Figures and Tables

**Table 1:** Physiochemical properties of raspberry ketone (RK).

**Chemical Structure**	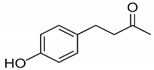
**Chemical Formula**	C_10_ H_12_ O_2_
**Molecular Weight**	164.204 g·mol^−1^
**Solubility**	ethanol, 1-propanol, 2-propanol, 1-butanol, 2-butanol, acetic acid, methyl acetate, ethyl acetate, acetone, and binary mixtures of ethanol + acetone [8]
**Melting Point**	81–85 °C (lit.)
**Boiling Point**	292.2 ± 15.0 °C at 760 mmHg
**Lambda Max**	280 nm
**LogP**	1.48

**Table 2:** Summary of therapeutic properties and mechanism of action of raspberry ketone (RK).

	Therapeutic Effect	Study Model	Mechanism of Action	Reference
1	Hepatoprotection	a. High-fat diet-fed Wistar rats b. Hepatotoxicity in male Wistar rats by CCL4 c. Acrylamide-induced hepatotoxicity in rats	↑TAC, PPAR-α, LDLR ↑TAC, GSH, SOD ↓AST, ALT, ALP, NF-κB, Caspase-3	[22,23,24]
2	Cardioprotection	a. Cardiotoxicity in Wistar albino rats by ISO b. Cardiotoxicity in Wistar albino rats by ISO	↑TAC, GSH, SOD, CAT ↓MDA ↑PPAR-α	[16,30]
3	Gastric Ulcers	Gastric lesions in male Wistar rats by EtOH	↑GSH px, GSH, CAT ↑Nrf2, ↓HMGB1	[41]
4	Depigmentation	a. Murine B16 Melanoma cells in vitro, zebrafish in vivo b. Biomimetic study	↓Cellular tyrosine, melanogenesis ↑Melanotoxicity	[3,46]
5	Anti-Obesity	a. High-fat diet-fed mice b. 3T3L1 murine adipose cells c. High-fat diet-fed Wistar rats d. Obese rats fed low-dose RK e. A clinical study in obese patients	↓Hepatic triglycerides ↑Lipolysis ↑Triglyceride catabolism, lipolysis ↑AQP7 ↓leptin ↓Reduced weight gain ↓Weight, metabolic, and lipid parameters	[5,10,11,56,58]
6	Bone Regeneration	C3H103T1/2 cells	↑Osteoblast, adipocyte differentiation	[70]

TAC, total antioxidant capacity; PPAR-α, peroxisome proliferator activating enzyme α; LDLR, low-density lipoprotein receptor; CAT, catalase; CCl_4_, carbon tetrachloride; NF-κB, nuclear factor-κB; EtOH, ethanol; GSH, glutathione; GSH-Px, glutathione peroxidase; HMGB1, high-mobility group box-1; ISO, isoproterenol; MDA, malondialdehyde; AST, aspartate aminotransferase; ALT, alanine aminotransferase; ALP, alkaline phosphatase; Nrf-2, nuclear factor erythroid-derived 2-related factor 2; SOD, superoxide dismutase; AQP-7, aquaporin-7. The up arrow (↑) indicates that the level or expression of each variable has increased, and the down arrow (↓) indicates a decrease in the corresponding value.

## Data Availability

Not applicable.
